# Evaluation of HRP2 and pLDH-based rapid diagnostic tests for malaria and prevalence of *pfhrp*2/3 deletions in Aweil, South Sudan

**DOI:** 10.1186/s12936-022-04280-w

**Published:** 2022-09-09

**Authors:** Emily Lynch, Tomas O. Jensen, Bachir Assao, Menard Chihana, Thadeous Turuho, Dan Nyehangane, John B. Manyok, Harriet Pasquale, Nimol Khim, Benoit Witkowski, Matthew E. Coldiron

**Affiliations:** 1 Epicentre, 14-34 Avenue Jean Jaurès, Paris, France; 2 Médecins Sans Frontières, 14-34 Avenue Jean Jaurès, Paris, France; 3 Center of Excellence for Health, Immunity, and Infections (CHIP) and Department of Infectious Diseases, Rigshospitalet, Copenhagen, Denmark; 4 Epicentre, Maradi, Niger; 5 Epicentre, Cape Town, South Africa; 6 Epicentre, Mbarara, Uganda; 7 Médecins Sans Frontières - France, Aweil, South Sudan; 8 National Ministry of Health, Juba, South Sudan; 9 Malaria Molecular Epidemiology Unit, Institut Pasteur du Cambodge, Phnom Penh, Cambodia

**Keywords:** Malaria, Rapid diagnostic test, Plasmodium lactate dehydrogenase, Histidine-rich protein 2, South Sudan

## Abstract

**Background:**

Rapid diagnostic tests (RDT) for malaria are the primary tool for malaria diagnosis in sub-Saharan Africa but the utility of the most commonly used histidine-rich protein 2 (HRP2) antigen-based tests is limited in high transmission settings due to the long duration of positivity after successful malaria treatment. HRP2 tests are also threatened by the emergence of *Plasmodium* that do not carry *pfhrp*2 or *pfhrp* 3 genes. *Plasmodium* lactate dehydrogenase (pLDH)-based tests are promising alternatives, but less available. This study assessed the performances of HRP2 and pLDH(pan) tests under field conditions.

**Methods:**

The study performed a prospective facility-based diagnostic evaluation of two malaria RDTs in Aweil, South Sudan, during the high transmission season. Capillary blood by fingerprick was collected from 800 children under 15 years of age with fever and no signs of severity. SD Bioline HRP2 and CareStart pLDH(pan) RDTs were performed in parallel, thick and thin smears for microscopy were examined, and dried blood was used for PCR testing.

**Results:**

Using microscopy as the gold standard, the sensitivity of both tests was estimated at  > 99%, but the specificity of each was lower: 55.0% for the pLDH test and 61.7% for the HRP2 test. When using PCR as the gold standard, the sensitivity of both tests was lower than the values assessed using microscopy (97.0% for pLDH and 96.5% for HRP2), but the specificity increased (65.1% for pLDH and 72.9% for HRP2). Performance was similar across different production lots, sex, and age. Specificity of both the pLDH and HRP2 tests was significantly lower in children who reported taking a therapeutic course of anti-malarials in the 2 months prior to enrollment. The prevalence of *pfhrp*2/3 deletions in the study population was 0.6%.

**Conclusions:**

The low specificity of the pLDH RDT in this setting confirms previous results and suggests a problem with this specific test. The prevalence of *pfhrp*2/3 deletions in the study area warrants continued monitoring and underscores the relevance of assessing deletion prevalence nationally. Improved malaria RDTs for high-transmission environments are needed.

**Supplementary Information:**

The online version contains supplementary material available at 10.1186/s12936-022-04280-w.

## Background

Because of their ease of use and relatively low price, rapid diagnostic tests (RDT) have become the first-line malaria diagnostic tests in most malaria-affected countries in sub-Saharan Africa. Current malaria RDTs are immunochromatographic tests that detect the presence of circulating parasite antigens, and their performance has been monitored by the World Health Organization (WHO), which runs a rigorous testing and quality assurance process [1]. The two most commonly targeted antigens are Histidine-rich protein 2 (HRP2), which is specific to *Plasmodium falciparum,* and *Plasmodium* lactate dehydrogenase (pLDH), which is present in all *Plasmodium* species; currently available tests detect one or both antigens. HRP2-based tests have been preferred in areas where *P. falciparum* is predominant, due to a higher reported sensitivity [2]. There is also evidence that they are more heat-stable than pLDH-based tests [3]. In vivo, the two antigens are cleared with different speeds, which appears to affect their specificity, and this is particularly important in high-transmission areas. An evaluation in Uganda showed that median time for an HRP2 test to become negative after an effective treatment was 35–42 days, but that the median time to become negative for a pLDH test was only 2 days, leading to a higher specificity of the pLDH in high-transmission areas [4, 5].

Because of the lower specificity of HRP2-based tests in high-transmission environments, which can lead to false positive results and overtreatment, pLDH-based malaria RDTs have been introduced in some settings, though supplies of pLDH-based tests are considerably lower than those of HRP2-based tests. Between 2016 and 2020, the international medical humanitarian organization Médecins Sans Frontières introduced the CareStart^™^ Malaria PAN (pLDH) Ag RDT (Reference RMNM-02571) in several high-transmission environments. In most of these settings, it replaced the previously used SD BIOLINE Malaria Antigen (Reference 05FK50). Both of these tests easily met overall WHO performance thresholds in their most recent evaluations, with good performance even at the lowest level parasitaemia [6]. Nonetheless, despite the high performance of the pLDH-based RDT in formal evaluations, a field-based evaluation in Niger suggested that its performance was virtually indistinguishable from that of the previously used HRP2-based RDT, with a specificity of only 57.4% (95%CI 51.5–61.3) compared to microscopy during the high transmission season [7]. Furthermore, in early 2020, a formal Notice of Concern was issued by WHO concerning the manufacture of the pLDH-based RDT [8].

Concerns about the accuracy of HRP2-based RDTs have arisen in recent years because of the increased prevalence of *Plasmodium* missing the *pfhrp*2 gene in multiple sub-Saharan African countries [9, 10]. In the case of *Plasmodium* that have deletions of both the *pfhrp*2 and *pfhrp*3 genes, the parasite is often undetectable by HRP2-based RDTs [11]. These double deletions have been more commonly reported in eastern Africa in recent years [12–14], but their presence has not yet been established in South Sudan.

To accompany its introduction in South Sudan, the study performed a formal clinical diagnostic evaluation of the newly introduced pLDH-based RDT, in parallel with the previously used HRP2-based RDT, in field conditions in Aweil, South Sudan. Off-site microscopy by at least two blinded microscopists was the gold standard comparator, and RDT performance was also compared to quantitative PCR. The presence of *pfhrp*2/3 deletions was investigated in all samples.

## Methods

### Study design and setting

Malaria transmission in northwest South Sudan has a marked seasonality, with a relatively long peak season that occurs between June and December, following the rainy season. In 2020, there were an estimated 3,211,331 cases and 7431 malaria deaths in South Sudan, including 457,888 cases in Northern Bahr el-Ghazal (NBeG) State, of which Aweil is the capital city [15].

**Fig. 1:**
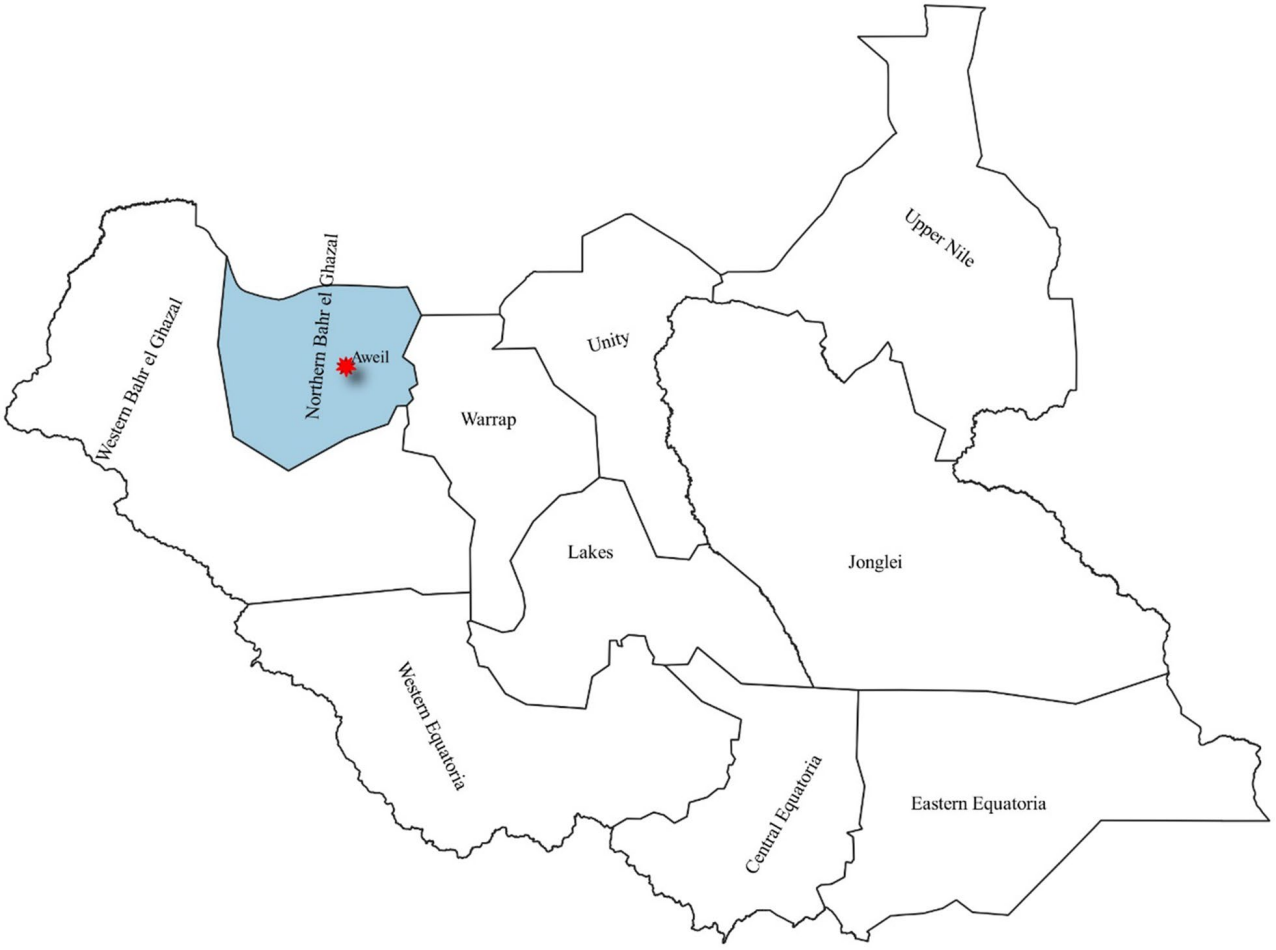
Northern Bahr el Ghazal State in South Sudan

The study planned to perform a prospective health centre-based, clinical diagnostic evaluation in two phases: first during the peak malaria transmission season (October–November 2019) and then during the low malaria transmission season (February–April 2020), but the low season phase was cancelled after the issuance of the Notice of Concern by WHO. The study was conducted in Aweil State Hospital, which is the referral hospital for NBeG (Fig. 1).

### Sample collection and processing

The study enrolled children aged between 3 months and 15 years presenting to the malaria-specific paediatric outpatient centre at Aweil State Hospital with fever (37.5 °C axillary temperature) or a history of fever in the preceding 48 h. Children from whom blood samples could not be safely obtained (i.e. low weight or severe anaemia) were excluded as were children with any sign of severity (including but not limited to decreased consciousness and seizure), who were referred immediately to the hospital emergency room for treatment. Because of logistic constraints in laboratory processing, enrollments were limited to the first 20 eligible children who presented each day.

After obtaining written informed consent from the participant’s caregiver and documenting oral assent for participants aged 7 years and older, a study nurse collected demographic and clinical information about each participant. This included age, sex, village of origin, whether the child slept under a bed net the previous night, receipt of intermittent preventive treatment for malaria in infants (IPTi) or curative anti-malarial treatment in the preceding 2 months, the date and time of the performance of the test, and the production lot number of the RDT.

The study nurse collected capillary blood by a fingerstick, preparing two thick and thin smears, and collecting 2–3 drops of blood directly onto filter paper. The study nurse wrote the patient ID number and time of testing on the RDT cassettes. The two tests (SD Bioline Ag P.f. (HRP2), Standard Diagnostics, Giheung-ku, Republic of Korea, catalog number 05FK50; and CareStart pLDH(pan), AccessBio, Somerset, NJ, USA, catalog number RMNM-02571) were performed following the manufacturer’s instructions, and the results were recorded in the study register. Following manufacturer’s instructions, the RDTs were stored at temperatures  < 30 °C between shipment and their arrival to the study site (confirmed by Logtag), where they were stored at temperatures  < 30 °C with a dedicated temperature monitor. Two different production lots of the HRP2-based test were used, but only one production lot of the pLDH-based RDT was available during the study period.

Any child with a positive result on either test (pLDH or HRP2) was treated, free of charge, following national protocols, with antipyretics, artemether-lumefantrine, and any other treatment deemed necessary by the treating clinician. Febrile children with a negative RDT were treated according to the clinician’s best judgment.

### Laboratory methods

At the end of each day, both slides were stained (10% Giemsa solution for 15 min), and the slide judged to be of lesser quality was discarded. Slides were stored and transported to the Epicentre laboratory in Mbarara, Uganda at the end of the study period, where microscopy was carried out according to WHO recommendations [16]. Microscopist competency was assessed with standard smears, verified with PCR, obtained from Shoklo Malaria Research Unit (SMRU) in Thailand. A total of 60 smears comprised of positives, negatives, all four *Plasmodium* species and mixed infections were used for this assessment. Microscopists were considered competent and allowed to read study smears if they scored  ≥ 90% in the proficiency assessment. In brief, the microscopists performed blind double-reading of all slides, with 200 high-power fields for negativity, and a third reading by a blinded microscopist in case of discrepant results between the first two for detection, species identification or parasitaemia discrepancy  > 50%. For positive slides, results included parasitaemia (asexual forms only) and species identification. The presence or absence of gametocytes was noted for all samples. Approximately 10% of negative and 10% of positive slides were sent for external quality control at the Shoklo Malaria Research Unit in Thailand.

Dried blood spots (DBS) were dried, wrapped and sealed in plastic bags according to standard operating procedures, stored at the study site and then sent to Institute Pasteur Cambodia (IPC) for further PCR analysis at the end of data collection period. Genomic DNA was extracted from the DBS using a DNA blood kit (Catalog 51306, Qiagen, Germany) according to manufacturer’s instructions. Presence of *Plasmodium* spp DNA was determined using real time PCR targeting the cytochrome b gene using previously-described methods [17]. Speciation (*Plasmodium falciparum, Plasmodium vivax, Plasmodium malariae, Plasmodium ovale)* was determined using specific real-time PCR [17, 18]. For samples identified as *P. falciparum* below a threshold of 30 cycles in the PCR, the presence or absence of deletion of *pfhrp*2 and *pfhrp*3 genes was determined by RT-PCR detecting parasite DNA. Reference strain DNA from Dd2, HB3 and 3D7 were used as controls of *pfhrp*2 deletion, *pfhrp*3 deletion, and absence of deletion, respectively. *Plasmodium falciparum* tubulin gene was used as a control for amplification. Primer sequences and the PCR conditions are available in Additional file 1.

### Statistical methods and sample size

The target sample size was calculated based on the number of true-positive and true-negative cases needed to estimate a sensitivity of 95% with a total width of the 95%CI of 8%, and a specificity ranging between 80% and 95% with a 95%CI width of 10.5% (for a point estimate of 80%) and 6% (for a point estimate of 95%). Under this scenario, it would be necessary to enroll a minimum of 140 true positives (e.g. children with malaria according to the gold standard) and 240 true negatives (e.g. children without malaria according to the gold standard). Based on historical data from Aweil Hospital, a 70% positivity rate among febrile children during the high season was assumed, and the study therefore aimed to enroll 800 children in Phase I (expecting 560 true-positives and 240 true-negatives).

The performance characteristics of each RDT (sensitivity, specificity, positive predictive value [PPV] and negative predictive value [NPV]) were calculated independently, and 95%CI were calculated using the exact binomial method. Only infections with *P. falciparum* (mono-infection or co-infection) asexual forms were considered positive for analytic purposes. The gold standard for the primary objectives was microscopy; pre-specified secondary analysis used qPCR as a gold standard. Pre-specified subgroup analysis included children  < 5, patients without recent history of anti-malarial treatment, and parasitaemia levels:  < 200, 200–1999, 2000–199999 and  ≥ 200000 parasites/µl by microscopy. Comparisons in these pre-specified subgroups should nonetheless be regarded as exploratory, as the target sample size was not set with the goal of making these comparisons. Data were analysed using Stata version 16 (College Station, TX, USA).

## Results

### Description of participants

A total of 800 participants were enrolled between 10 October and 4 December 2019, including 471 (59%) with *P. falciparum* parasitaemia by microscopy. A total of 792 participants (99%) reported sleeping under a bed net the night prior to enrollment, and 259 (32%) reported having taken an anti-malarial drug in the preceding 2 months, including 168 (21%) who had taken it in the 30 days prior to enrollment and 74 (9%) in the 7 days prior to enrollment. No participant reported having received IPTi. Full results of microscopy and PCR are presented in Table 1. Among cases with *P. falciparum* parasitaemia by microscopy, 459 were mono-infections, 11 had *P. falciparum/P. malariae co-*infection, and 1 had mixed *P. falciparum/P. ovale* co-infection. Parasite density did not differ by age group (Kruskal–Wallis p = 0.36). A total of 542 samples (68%) were positive for *P. falciparum* by PCR, including 30 which had co-infection with at least one other *Plasmodium* species (Table 1).

**Table 1:** Demographic characteristics of participants and description of malaria parasitaemia, Aweil, South Sudan, 2019 (n = 800)

Characteristic	
Sex, n (%)	
M	446 (56)
F	354 (44)
Age in years	
Under 5, n (%)	505 (63)
5–14, n (%)	295 (37)
Median age in years (IQR)	3 (1–7)
*P falciparum* parasitaemia by microscopy n (%)	471 (59)
Median parasitaemia (IQR), asexual parasites/µl	38446 (6886–86215)
< 200/µl, n (%)	28 (5.9)
200–1999/µl, n (%)	51 (10.8)
2000–199999/µl, n (%)	367 (77.9)
≥ 200000/µl, n (%)	25 (5.3)
*P falciparum* gametocytemia by microscopy, n (%)	93 (11.6)
*P falciparum* parasitaemia by PCR n (%)	542 (68)
* P. falciparum* mono-infection	512 (94.5)
* P. falciparum* and *P. malariae*	20 (3.7)
* P. falciparum* and *P. ovale*	3 (0.6)
*P. falciparum* and *P. vivax*	1 (0.18)
* P. falciparum* and *P. malariae* and *P. vivax*	5 (0.9)
* P. falciparum* and *P. malariae* and *P. ovale*	1 (0.18)

### Performance characteristics of RDTs

For the pLDH test, there were 616 positive results and 184 negative results. For the HRP2 test, there were 593 positive results and 207 negative results (Table 2). Using microscopy as gold standard, the sensitivity of the pLDH test was 99.4% and that of the HRP2 test was 99.2%; the specificity of the pLDH test was 55.0% and that of the HRP2 test was 61.7% (Table 3). When compared to PCR, the sensitivity of the pLDH test was 97.0%1 and that of the HRP2 test was 96.5%; the specificity of the pLDH test was 65.1% and that of the HRP2 test was 72.9%. The point estimates of PPV for both tests were similar (76–78% with microscopy as gold standard and 85–88% with PCR as gold standard). The estimates of NPV were also similar between the two tests, though the NPV was higher when microscopy was used as gold standard (98%) than when PCR was used as the gold standard (90–91%).

**Table 2:** Results of HRP2 and pLDH RDTs for individual samples, Aweil, South Sudan, 2019

pLDH	HRP2		Total
	Positive	Negative	
Positive	590	26	616
Negative	3	181	184
Total	593	207	800

**Table 3:** Performance characteristics of two malaria rapid diagnostic tests, Aweil, South Sudan, 2019^*^

Characteristic test	Microscopy as gold standard			PCR as gold standard		
	N^†^	Value	95% CI	N^†^	Value	95% CI
Sensitivity						
pLDH	471	99.4	98.1–99.9	542	97.0	95.3–98.3
HRP2	471	99.2	97.8–99.8	542	96.5	94.6–97.9
Specificity						
pLDH	329	55.0	49.5–60.5	258	65.1	59.0–70.9
HRP2	329	61.7	56.2–67.0	258	72.9	67.0–78.2
PPV						
pLDH	616	76.0	72.4–79.3	616	85.4	82.3–88.1
HRP2	593	78.8	75.2–82.0	593	88.2	85.3–90.7
NPV						
pLDH	184	98.4	95.3–99.7	184	91.3	86.3–94.9
HRP2	207	98.1	95.1–99.5	207	90.8	86.0–94.4

^*^PPV positive predictive value, *NPV* negative predictive value

^†^For sensitivity, N represents all true positives by microscopy or PCR; for specificity, N represents all true negatives by microscopy or PCR; for PPV, N represents all RDT test positives; and for NPV, N represents all RDT test negatives

The sensitivity of the pLDH test was 100% at all except the lowest parasite densities (< 200 parasites/µl), as judged by microscopy. The sensitivity of the HRP2 test was above 99% at parasite densities  > 2000 parasites/µl, and 98% at parasite densities between 200 and 1999 parasites/µl (Table 4). Nonetheless, it should be noted that the confidence intervals around the lower parasite density estimations are much wider, as there were fewer positive results. The specificity of both the pLDH and HRP2 tests was below 50% among participants reporting having taken an anti-malarial treatment in the 2 months prior to enrollment (Table 5).

**Table 4:** Sensitivity of two malaria RDTs by parasite density, Aweil, South Sudan, 2019

Test parasites/µl	Microscopy as gold standard		
	N with given parasitaemia	Value	95% CI
pLDH	471	99.4	98.1–99.9
< 200	28	88.9	70.8–97.6
200–1999	51	100	–
2000–199999	367	100	–
≥ 200000	25	100	–
HRP2	471	99.2	97.8–99.2
< 200	28	88.9	70.8–97.6
200–1999	51	98.0	89.6–100
2000–199999	367	100	–
≥ 200000	25	100	–

**Table 5:** Performance characteristics of two malaria rapid diagnostic tests, stratified by recent treatment for malaria, Aweil, South Sudan, 2019^*^

	Malaria treatment in prior 2 months	Microscopy as gold standard			PCR as gold standard		
		N^*^	Proportion	95% CI	N^*^	Proportion	95% CI
Sensitivity							
pLDH	No	318	99.1	97.3–99.8	356	96.3	93.8–98.0
	Yes	153	100	97.6–100	186	98.4	95.4–99.7
HRP2	No	318	98.7	96.8–99.7	356	95.5	92.8–97.4
	Yes	153	100	97.6–100	186	98.4	95.4–99.7
Specificity							
pLDH	No	225	65.8	59.2–72.0	187	73.8	66.9–79.9
	Yes	104	31.7	22.9–41.6	71	42.3	30.6–54.6
HRP2	No	225	72.9	66.6–78.6	187	81.3	74.9–86.6
	Yes	104	37.5	28.2–47.5	71	50.7	38.6–62.8

^*^For sensitivity, N represents all true positives by microscopy or PCR; for specificity, N represents all true negatives by microscopy or PCR

There were no differences in performance characteristics of the test by participant sex, age group, or production lot of the RDT used. Overall, of the 542 samples with *P. falciparum* parasitaemia by PCR, 469 (86%) had a cycle threshold (CT)  < 30 and could, therefore, be reliably analysed for the presence of *pfhrp*2/3 deletions. One sample had a single *pfhrp*2 deletion, one sample had a single *pfhrp*3 deletion, and three samples (0.6%) had double *pfhrp*2/3 deletions. The *pfhrp*2 RDT was positive for 2 of the 3 samples with double *pfhrp*2/3 deletions, as well as for both samples with a single deletion (Table 6).

**Table 6:** Description of participants with *pfhrp*2/3 deletions (n = 5), Aweil, South Sudan, 2019

Age	Sex	Presence of *pfhrp*2/3 genes	HRP2 RDT result	pLDH RDT result	Microscopy result	Parasite density by microscopy (parasites/µl)	Speciation by PCR	*P. falciparum* cycle threshold
5y	M	*hrp*2−/*hrp*3−	Positive	Positive	Negative	–	*P. falciparum*	28.18
8y	M	*hrp*2−/*hrp*3−	Negative	Positive	*P. malariae*	1452	*P. falciparum* *P. malariae* *P. vivax*	24.32
1y	F	*hrp*2−/*hrp*3-	Positive	Positive	*P. falciparum*	311	*P. falciparum*	27.37
9 m	M	*hrp*2−/*hrp*3 +	Positive	Positive	*P. falciparum*	29744	*P. falciparum*	22.01
8y	F	*hrp*2 + /*hrp* 3−	Positive	Positive	*P. falciparum* *P. malariae*	3538	*P. falciparum* *P. malariae*	23.88

It is important to note that of the 26 samples positive by pLDH RDT but negative by HRP2 RDT (Table 2), 18 were negative by both microscopy and PCR. *Plasmodium malariae* was detected by microscopy and/or PCR (either as monoinfection or mixed infection) in 7 of the samples. *Plasmodium falciparum* was detected in 3 of those samples (one mono-infection, two mixed infections), with only one sample having a CT  < 30. That sample did indeed have *pfhrp*2/3 deletions and is shown in the second line of Table 6.

## Discussion

As seen in a previous study in Niger, when using microscopy as a gold standard the specificity and PPV of the pLDH RDT were low, and very similar to those of the HRP2 test [7]. At the time of the study implementation, seasonal malaria chemoprevention (SMC), which was reported as a potential explanation for poor specificity in Niger, had not been implemented in South Sudan. The performance of both the pLDH and HRP2 RDTs slightly improved when using PCR as the gold standard, seemingly because of improved detection of low-density parasitaemia. Nonetheless, the study findings remain potentially problematic for the pLDH test under consideration, all the more so considering the 2020 WHO statement of concern [8] regarding its production quality. To further understand test performance it would also be informative to conduct this study during the low-transmission season in Aweil, or in other settings with lower malarial endemicity.

The prevalence of parasitaemia (73%) among febrile children presenting for care was in line with expectations and was higher than in other evaluations performed in areas receiving chemoprevention programs like SMC (at the time of this study, SMC had never been administered in the study area). The decreased specificity of the pLDH and HRP2 test among children who had received anti-malarials underscores the known limitations of HRP2 tests in high transmission environments, where persistent antigenaemia after successful treatment leads to diagnostic and therapeutic dilemmas for treating clinicians. pLDH-based RDTs are expected to mitigate these problems, but that effect was not seen in this setting, nor in the Niger study.

This study used expert microscopy as the primary gold standard for evaluating RDT performance. As a secondary objective, RDT performance was evaluated against PCR, which should have a lower limit of detection. The main results suggest that this was indeed the case. The decreased sensitivity and NPV of both RDTs when PCR was used as the gold standard likely suggests that low or very low-level parasitaemia was responsible for most false negative results. The clinical implications of this are unclear, particularly given the high prevalence of malaria among febrile children seeking care in the study area, as well as their overall high parasite densities, though it is plausible that among children with very low densities their fevers were not malarial in origin [19, 20].

Data on the prevalence of *pfhrp*2/3 deletions in South Sudan is scarce. To consider a national switch to RDTs based on another antigen (pLDH, aldolase, or combination HRP2/pLDH tests), the WHO recommends a threshold of ≥5% prevalence of *pfhrp*2/3 deletions among pfHRP RDT suspected false negatives confirmed by either pLDH RDT or microscopy among clinical cases in one location (including 370 samples from at least 10 facilities in site) [21]. This current study was not designed to provide an estimate of deletion prevalence according to this sampling method. However, as the study detected minimal circulation of parasites with the double *pfhrp*2/3 deletion and given the high prevalence of deletions seen in the nearby Horn of Africa, the authors consider that the 0.6% prevalence seen in this study population supports increased vigilance in South Sudan. Further, it is curious that 2 of the 3 samples with the double *pfhrp*2/3 deletion were detected by the *pfhrp*2 RDT. The authors do not have a simple explanation for this unexpected result, though one possibility is multiple *P. falciparum* strains present in one sample, with persistent antigenaemia from a recent malaria episode from a strain without the *pfhrp*2/3 deletions leading to both a positive HRP2 RDT as well as a finding of double *pfhrp*2/3 deletion in that same sample.

This study has certain limitations. One is that the study did not obtain data about the specific timing of the use of curative anti-malarials in the time before presentation. Second, this study reports only on the RDT performance during the high transmission season, when parasite prevalence is at its highest; overall performance of both RDTs may be different in the low season, when parasite densities and overall prevalence tend to be lower.

## Conclusions

This evaluation was undertaken to support the introduction of the pLDH RDTs in South Sudan, but the results showed specificity that was essentially the same as that seen with HRP2-based RDTs, similarly to the results seen in the Niger study. The ensuing overtreatments were not ideal, particularly given that the higher specificity of this more costly pLDH test was supposed to be its advantage over traditional HRP2 tests in high transmission environments. The prevalence of *pfhrp*2/3 deletions cannot be interpreted in the context of a national recommendation, but this area of concern should be followed closely both in NBeG and in other areas of South Sudan. The study findings support the continued use of the HRP2 RDT for routine malaria diagnosis in South Sudan until such point as a pan-pLDH RDT is developed that both performs well and can be feasibly incorporated into the national malaria strategy guidelines.

## Supplementary Information

**Additional file 1. **Primer sequences and PCR conditions for PCR testing of study samples at Institute Pasteur Cambodia.

## Data Availability

The datasets generated and analysed during this study are available from the corresponding author on reasonable request.

## Abbreviations

ACT: Artemisinin-based combination therapy
HRP2: Histidine-rich protein 2
HRP3: Histidine-rich protein 3
IPTi: Intermittent preventive treatment for malaria in infancy
NPV: Negative predictive value
PCR: Polymerase chain reaction
pLDH: *Plasmodium* lactate dehydrogenase
PPV: Positive predictive value
RDT: Rapid diagnostic test
SMC: Seasonal malaria chemoprevention
WHO: : World Health Organization
