# Supplementary material for: Evaluation of HRP2 and pLDH-based rapid diagnostic tests for malaria and prevalence of pfhrp2/3 deletions in Aweil, South Sudan
Source: Malar J. 2022 Sep 9;21:261. doi: 10.1186/s12936-022-04280-w (PMC9461093; doi:10.1186/s12936-022-04280-w)
Supplement: Supplementary file 1 — Additional file 1. Primer sequences and PCR conditions for PCR testing of study samples at Institute Pasteur Cambodia. [file 12936_2022_4280_MOESM1_ESM.docx]

| **Gene ID** | **Primer name** | **Sequence (5'-3')** | **Master mix** | **Assay parameters** | **Melt parameters** | **Melting Tm peak** | **Expected product size** |
| --- | --- | --- | --- | --- | --- | --- | --- |
|  |  |  |  |  |  |  |  |
| histidine-rich protein II  (PF3D7_0831800) | Hrp-2_New_2F (exon2) | ATAATTCCGCATTTAATAATAACTTGTGT | Hot FirePol EvaGreen qPCR Mix,  Solis Biodyne 1X (#08-24-00020), Primers 150 nM, 5 μl DNA template,  Total volume  20 μl | 1 cycle: 95°C-15 min  45 cycles: 95°C-15 sec/ 60°C-63^0^C* -20sec/ 72°C-20 sec  95°C-2min 68°C-2 min | From 68 to 90°C, increment 0.2°C for 0.05 sec | 75.0-75.4^0^C | 100 bp |
|  | Hrp-2_New_2R (exon2) | GCATCATCTACATGTGCTTGAGTT |  |  |  |  |  |
| histidine-rich protein III (PF3D7_1372200) | Hrp-3_New_2F (exon2) | GCTGATGCTAATCACGGATTTC |  |  |  | 78.4-78.8^0^C | 108 bp |
|  | Hrp-3_New_2R (exon2) | GGCATCGTCATGGTGAGAAT |  |  |  |  |  |
| Plasmodium falciparum tubulin beta chain (PF3D7_1008700) | Pftub_1F | TGATGTGCGCAAGTGATCC |  |  |  | 78.8-79.2^0^C | 79 bp |
|  | Pftub_1R | TCCTTTGTGGACATTCTTCCTC |  |  |  |  |  |

^*63°C: annealing temperature for RT-PCR Pftubulin^
